# LmRaC: a functionally extensible tool for LLM interrogation of user experimental results

**DOI:** 10.1093/bioinformatics/btae679

**Published:** 2024-11-15

**Authors:** Douglas B Craig, Sorin Drăghici

**Affiliations:** Department of Emergency Medicine Research, Michigan Medicine, University of Michigan, Ann Arbor, MI 48109, United States; Department of Computer Science, Wayne State University, Detroit, MI 48202, United States

## Abstract

**Motivation:**

Large Language Models (LLMs) have provided spectacular results across a wide variety of domains. However, persistent concerns about hallucination and fabrication of authoritative sources raise serious issues for their integral use in scientific research. Retrieval-augmented generation (RAG) is a technique for making data and documents, otherwise unavailable during training, available to the LLM for reasoning tasks. In addition to making dynamic and quantitative data available to the LLM, RAG provides the means by which to carefully control and trace source material, thereby ensuring results are accurate, complete, and authoritative.

**Results:**

Here, we introduce LmRaC, an LLM-based tool capable of answering complex scientific questions in the context of a user’s own experimental results. LmRaC allows users to dynamically build domain specific knowledge-bases from PubMed sources (*RAG_dom_*). Answers are drawn solely from this RAG with citations to the paragraph level, virtually eliminating any chance of hallucination or fabrication. These answers can then be used to construct an experimental context (*RAG_exp_*) that, along with user supplied documents (e.g. design, protocols) and quantitative results, can be used to answer questions about the user’s specific experiment. Questions about quantitative experimental data are integral to LmRaC and are supported by a user-defined and functionally extensible REST API server (*RAG_fun_*).

**Availability and implementation:**

Detailed documentation for LmRaC along with a sample REST API server for defining user functions can be found at https://github.com/dbcraig/LmRaC. The LmRaC web application image can be pulled from Docker Hub (https://hub.docker.com) as dbcraig/lmrac.

## 1 Introduction

Top scientific journals like Nature have recognized the potential of large-language models (LLMs) such as OpenAI’s GPT-4 to change how science is done. In particular, LLMs and their precursors (Beltagy *et al.* 2019, Lee *et al.* 2020, Gu *et al.* 2021, Luo *et al.* 2022) have rapidly moved toward human levels of competence in biology. However, as performance has grown, so too have concerns about their limitations and faults. Scientists reviewing LLMs have raised ethical concerns, noted a lack of transparency, and, more troubling, worried about the generation of fake “facts” (Sanderson 2023). In fact, posing this concern to GPT-4o, OpenAI’s latest release, yields this insightful warning:

Users may sometimes mistake the generated text as authoritative answers rather than outputs of a probabilistic model… It is crucial for users to understand that these answers are generated based on patterns rather than verified knowledge, and they should not be blindly trusted as authoritative or factually accurate.

Undermining the warning, however, is the well documented phenomenon of hallucinations (Huang *et al.* 2023, Zhang *et al.* 2023). For though we’ve been warned not to blindly trust, LLMs routinely fabricate authoritative citations in an effort to convince us otherwise. Compounding this problem is the tendency of users to conflate training with recall leading them to treat LLMs as stores of knowledge. LLMs are *not* databases. Though trained on billions of pieces of text, they are not explicitly designed to accurately recall the text they’ve been trained on, much less cite its source. Scientific research depends on both.

One approach to addressing these concerns is to leverage an LLM’s strength for language reasoning, while severely limiting its use as a reliable knowledge source (Truhn *et al.* 2023). Instead, knowledge *external* to the LLM can be made readily available using retrieval-augmented generation (RAG) techniques (Lewis *et al.* 2021). This has the added benefit that this external knowledge need not be limited to static documents. Meanwhile, the LLM is used solely as a reasoning engine to compare, summarize, extrapolate and evaluate from this external information thus removing the LLM as a primary knowledge source.

We take that approach here and introduce Language Model Research Assistant & Collaborator (LmRaC), pronounced “Limerick” (as in the verse form and Irish county). LmRaC is designed to support researchers by incrementally building custom knowledge bases from the scientific literature and seamlessly integrating it with knowledge of a user’s own experimental results thereby aiding them both in interpretation of their work as well as putting it in the larger context of current scientific knowledge. This can be especially helpful in differential gene expression experiments, for which LmRaC was initially designed, where hundreds of entities interact at multiple levels of abstraction (i.e. molecular, regulatory, pathway, cellular, organism).

Other recent work in science and medicine has shown RAG approaches have fewer hallucinations when answering questions about disease (Soong *et al.* 2023) and improve the accuracy and reliability of answers to clinical questions (Zakka *et al.* 2024). PaperQA is able to search PubMed and arXiv, evaluate papers and synthesize an answer (with references) using a RAG agent architecture (Lála *et al.* 2023). LmRaC moves beyond this work by offering accurate, reliable and complete answers that are traceable via citations down to the paragraph level while extending the RAG architecture to include a user’s own experimental data.

LmRaC is freely available as a downloadable web application and can be easily extended to integrate additional data and knowledge sources with its core functionality. This enables LmRaC to serve as a powerful extensible tool for researchers, providing detailed and accurate answers while being fully data-aware.

## 2 Materials and methods

LmRaC is a web application running as a Docker container. The base application focuses on answering scientific questions about genes, biological pathways and disease by building user directed domain specific knowledge bases. LmRaC also includes integral support for a user-defined REST API server to facilitate easy and flexible interface with user experimental data.

LmRaC employs a novel multi-tiered retrieval-augmented generation (RAG) design. Three distinct but interlinked RAGs ensure answers are reliably derived *only* from PubMed articles (https://pubmed.ncbi.nlm.nih.gov/), user documents and user data.

### 2.1 Domain specific knowledge bases

Domain-specific knowledge is dynamically generated as users ask questions. Given a question, LmRaC uses a controlled vocabulary of gene names, pathways, and diseases as well as an LLM generated general query to search PubMed for related journal articles. The retrieved articles are then chunked into paragraphs and vector embeddings calculated for each chunk. These embeddings are stored in a vector database along with associated metadata (i.e. gene, pathway or disease identifiers, paragraph MD5) for efficient searching. Users can segregate knowledge into separate databases, called indexes, as they see fit. These indexes constitute the domain knowledge RAG, or *RAG_dom_*.

### 2.2 Sub-question generation

Once domain knowledge has been collected for a question, the question is broken into sub-questions to improve the detail and thoroughness of the response. Sub-questions are constructed with database query in mind and then answered independently. The user can adjust the number of sub-questions depending on the complexity of the question and desired detail of the final answer.

### 2.3 Vector database query and paragraph usefulness assessment

Each sub-question in turn is converted into a vector embedding and queried against the vector database. Two types of queries are performed: a domain-wide search and a metadata-filtered search. This dual-query approach facilitates knowledge discovery from both labeled and unlabeled sources, and ensures that even terms not explicitly searched for, or included in the vocabularies, can be found. The combined set of best matches constitutes candidate paragraphs for answering the sub-question.

Candidate paragraphs are retrieved from PubMed and then evaluated by the LLM for their usefulness in answering the sub-question. Paragraphs with a score of 7 or higher (on a 10 point scale) are deemed high-quality and retained for the next step.

### 2.4 Answer generation and quality assessment

The set of high-quality paragraphs are provided to the LLM along with the sub-question and an answer is generated. Note that the LLM is explicitly disallowed through usefulness pre-screening and prompt engineering from using any information other than the supplied paragraphs to maximize the likelihood of a hallucination free answer that is traceable to paragraphs from identified primary sources. The quality of each sub-question answer is then assessed by the LLM as a further check before inclusion in the final combined and edited answer. Citations to all paragraphs used in answers are included as hyperlinks and a cumulative bibliography added to the final answer.

### 2.5 Question and answer with experimental context

Beyond question and answer, LmRaC also allows users to create named experimental contexts of data and documents. Like general domain knowledge, information in experiments is indexed. Documents relevant to an experiment may be uploaded by the user (e.g. background information, protocols, notes) or saved directly from previous LmRaC answers. In either case documents are chunked, embeddings computed and a searchable index created. These indexes constitute the experiment RAG, or *RAG_exp_*.

When users ask questions, they can explicitly specify whether to use general knowledge (*RAG_dom_*), experimental context (*RAG_exp_*), or both. LmRaC can also infer what knowledge to use based on the question, enabling it to follow a chain of reasoning that integrates general information with experimental context. This is a double-edged sword. While LmRaC minimizes hallucinations by using RAG, users are cautioned to avoid injecting misinformation into the experimental context which could result in factually incorrect answers. This technique was purposely used during testing to confirm that the LLM’s knowledge did not “leak” into answers.

### 2.6 Quantitative experimental results

Experimental results, typically structured or tabular data from analysis or measurement devices, can also be stored as part of an experiment. This information is made available for question answering through user-defined functions made accessible to LmRaC through an integrated REST API. This enables LmRaC to request and retrieve specific data as needed to answer user questions. This functional layer is referred to as the *RAG_fun_*. Base functionality for this REST API server is provided along with complete instructions for extending its functionality to make any user data type available to LmRaC.

### 2.7 Software implementation

LmRaC is implemented in Python 3. OpenAI’s *gpt-4o* model is used as a language reasoning engine through its API (https://platform.openai.com). Vector embeddings for both PubMed articles and experiment documents are calculated using the *text-embedding-ada-002* model with cosine distance. Vectors are stored in Pinecone serverless indexes (https://www.pinecone.io/). Full-text paragraphs are retrieved on-demand from PubMed (https://pmc.ncbi.nlm.nih.gov/tools/oa-service/) and their MD5s checked against embedding metadata. All XML documents are parsed using Beautiful Soup (https://www.crummy.com/software/BeautifulSoup/).

## 3 Results

**Figure 1.**
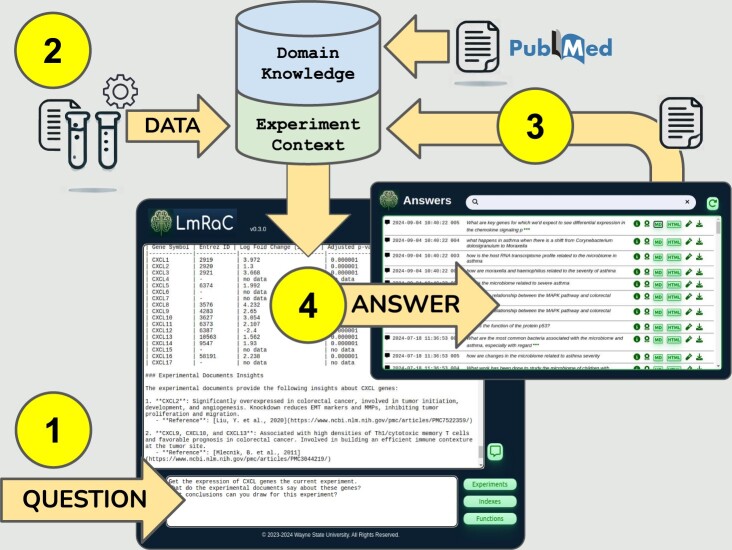
LmRaC workflow overview. (1) A user asks a question through the web application interface. This initiates a search of PubMed for articles relevant to mentioned genes, pathways, and/or diseases. These articles are saved as domain knowledge. (2) Quantitative data needed to answer the question is retrieved through a user-defined REST API as part of the experimental context. (3) Answers from previous questions may be saved to extend and refine the experimental context. (4) LmRaC answers the question using available domain knowledge and experimental context.

LmRaC workflow is summarized in Fig. 1. The following sections walk through analysis of a differential gene expression experiment using data from a comprehensive colorectal cancer study (The Cancer Genome Atlas Network 2012).

### 3.1 Creating indexes by asking questions

Users choose an existing or create a new index before asking any question. An index, stored in the Pinecone vector database, acts as a cumulative repository of information for answering questions in a particular domain. Each index is divided into two sections: primary source material from PubMed (*RAG_dom_*), and secondary material associated with one or more experiments (*RAG_exp_*). PubMed searches are limited to free full text articles published in the last 5 years. Secondary material may include any user provided documents, including: saved answers, protocols, and background knowledge relevant to the experiment. This dual-index approach allows LmRaC to provide comprehensive, data-aware answers to complex biological questions by integrating both authoritative general and experiment-specific knowledge and results.

OpenAI’s GPT-4o is used as a language reasoning engine only, not as a knowledge source. LmRaC is designed to answer questions based solely on its accumulated domain knowledge derived from PubMed journal articles and user provided experimental documents and results.

When a user asks a question, LmRaC analyzes it to identify any gene, biological pathway, and/or disease terms. Terms are assigned universal identifiers using vocabulary files derived from authoritative sources: HGNC (https://www.genenames.org/), KEGG (https://www.genome.jp/kegg/pathway.html) and MeSH (https://www.ncbi.nlm.nih.gov/mesh/). For example, in this experiment the user may ask a general question about expected gene expression in a particular pathway for the disease under study.

[user] What are key genes for which we’d expect to see differential expression in the chemokine signaling pathway in colorectal cancer?

The chemokine signaling pathway (KEGG hsa04062) and colorectal cancer (MeSH D015179) are identified. No specific genes are mentioned. Initial population of the index is then made by searching PubMed articles for these terms. In the case of pathways the search is based on primary articles cited for each KEGG pathway plus subsequent citations of these primary sources. Finally, LmRaC generates a general PubMed query for the whole question which finds articles that mention both the pathway and the disease. When searching, the user specifies how many of the most recent articles, for which full-text is available, should be loaded into the index. Vector embeddings (dim: 1536, metric: cosine) are computed for all article paragraphs and added to the index along with associated metadata (article, gene, pathway, disease identifiers). Subsequent questions check if information for terms is already part of the index and the user is given the option to search again for more recent material.

Although it is tempting to populate an index with as much information as possible, testing has shown that because PubMed searches are sorted by date and most scientific articles include reviews of previous and related work, often 10 or fewer articles for an item (e.g. gene) are needed to answer even moderately complex questions.

All answers are saved and available through the Answers dialog. Each answer is time stamped and summarized along with LmRaC’s quality assessment. Answers can be viewed as HTML or Markdown through a web browser as well as downloaded as plain text.

### 3.2 Experimental context and data-aware questions

Key to LmRaC’s design, answers can be fed back into the *RAG_exp_* of the user’s knowledge base to create an experimental context. This has the effect of focusing the answers to experimental questions. These answers can then be used to inform subsequent literature searches that can themselves further refine the context. This feature not only facilitates the interpretation of experimental data but also builds perspective for how new findings might fit with current scientific knowledge.

Continuing our example, the answer to the previous question regarding expected expression can be saved to the experiment. The user can now ask a question that uses this general information to answer questions about experimental results. For example:

**[user] Get the expression of CXCL genes in the experiment. What do the experimental documents say about these genes? What conclusions can you draw for this experiment?**

LmRaC answers by listing the measured expression of all CXCL genes in the user experiment, summarizing what the previously saved answer says about these genes in general and then interpreting the results in light of this information (answer edited for readability):

**[LmRaC] Gene Expression of CXCL Genes in the Experiment**

**CXCL1 (2919), logFC=3.972,*P*=0.000001**

**CXCL2 (2920), logFC=1.300,*P*=0.000001**

**…**

**Experimental Documents Insights**

**1. CXCL2: Significantly overexpressed in colorectal cancer, involved in tumor initiation, development, and angiogenesis. Knockdown reduces EMT markers and MMPs, inhibiting tumor proliferation and migration.**

**Reference: Liu *et al.* (2020)**https://www.ncbi.nlm.nih.gov/pmc/articles/PMC7522359/

**…**

**Conclusions for the Experiment**

**1. Overexpression of CXCL Genes: Several CXCL genes (e.g. CXCL1, CXCL3, CXCL8) show significant overexpression, indicating their potential role in promoting tumorigenesis and cancer progression.**

**2. Immune Response and Prognosis: CXCL9, CXCL10, and CXCL13 are linked to favorable prognosis due to their role in enhancing the immune response at the tumor site, suggesting their potential as biomarkers for immune contexture in cancer.**

**3. Therapeutic Targets: The involvement of CXCL2 and CXCL16 in cancer progression and metastasis highlights their potential as therapeutic targets. Inhibiting these chemokines could reduce tumor growth and spread.**

**4. Negative Regulation: CXCL12 shows a negative log fold change, suggesting it may have a different role or be downregulated in the context of this experiment.**

In this case LmRaC utilizes the *RAG_fun_* to treat questions about data, in this case CXCL gene expression, very much like any other question. Functions describe what “question” they can answer. LmRaC then seamlessly uses these descriptions to request the needed information.

From an implementation perspective, LmRaC acts as a client making requests to a REST API server. Function descriptions (along with descriptions of any required parameters, e.g. experiment name) are read in when LmRaC starts up. Before asking a question the user asks for the function to be loaded. This makes the function description available when answering subsequent questions. The GTP-4o API supports functions by passing an optional JSON description with any completion request. A complete working example for retrieving differential gene expression results (DEGbasic) is included in the LmRaC GitHub repository along with code for the REST API server. Full details for declaring and writing functions are also included.

### 3.3 Complex questions

LmRaC uses GPT-4o to rephrase questions into multiple sub-questions. It also implicitly provides a framework for answering multi-part questions iteratively. However, LmRaC is currently limited by this approach. Multi-part questions are effectively evaluated serially with each iteration using available loaded functions to gather missing information (e.g. expression of a mentioned gene). This places the burden on the user to ask questions that can be answered as a linear accumulation of information. To address this limitation ongoing LmRaC research is focused on nonlinear, multi-step problem solving with interactive user feedback and collaboration.

## 4 Conclusions

LmRaC offers researchers a dynamic tool for exploring and understanding complex biological phenomena. Its ability to seamlessly incorporate experimental data into its answers ensures that responses are not only accurate but also highly relevant to a specific research context. LmRaC’s built-in ability to make requests via a REST API also gives users the opportunity to significantly extend functionality to include a wide variety of data and knowledge sources. This makes LmRaC a valuable tool for researchers looking to gain deeper insights from their experiments and the broader scientific literature.

## Software availability

Detailed documentation for LmRaC along with a sample REST API server fordefining user functions can be found at https://github.com/dbcraig/LmRaC. The LmRaC web application image canbe pulled from Docker Hub (https://hub.docker.com) as dbcraig/lmrac.
